# Case Report: Ultrasound-guided continuous catheter drainage to facilitate ovarian access before *in vitro* fertilization in a patient with large pelvic cystic lesions

**DOI:** 10.3389/fmed.2026.1946025

**Published:** 2026-09-22

**Authors:** Kaiyue Zhang, Hualing Zhang, Xiuming Tang, Jianxin Liu, Yufeng Li, Rui Chen

**Affiliations:** Department of Reproductive Medicine, The Affiliated Hospital of Qingdao University, Qingdao, Shandong, China

**Keywords:** case report, *in vitro* fertilization, pelvic adhesive disease, pelvic cystic lesions, secondary infertility, ultrasound-guided drainage

## Abstract

Severe pelvic adhesive disease and large pelvic cystic lesions may markedly distort pelvic anatomy and compromise ovarian visualization and accessibility during *in vitro* fertilization (IVF). We report a 35-year-old woman with secondary infertility, bilateral tubal occlusion, severe pelvic adhesive disease, and large multiloculated pelvic cystic lesions that obscured the left ovary and displaced the right ovary superiorly. The lesions were clinically considered to represent chronic inflammatory pelvic sequelae, although their precise etiology remained uncertain. Before ovarian stimulation, transabdominal ultrasound-guided continuous drainage using a 12-Fr pigtail catheter was performed primarily to restore ovarian access. Approximately 450 mL of clear, odorless fluid was drained over approximately 10 days, reducing the dominant lesion from 18.40 cm × 8.82 cm × 11.80 cm to approximately 3 cm × 2 cm. Following drainage, the number of sonographically visualized antral follicles in the ovary increased from two to eight, reflecting improved visualization rather than increased ovarian reserve, and the previously obscured left ovary became accessible. Five metaphase II oocytes were retrieved, four from the left ovary and one from the right ovary; four high-quality blastocysts were vitrified, and a live birth followed a single frozen-thawed blastocyst transfer. In this patient, continuous catheter drainage provided temporary mechanical decompression that facilitated ovarian access and IVF treatment. This single case cannot establish the efficacy, safety, or generalizability of this strategy.

## 1 Introduction

Pelvic inflammatory disease (PID) can lead to tubal occlusion, hydrosalpinx, pelvic adhesions, and infertility, although its etiology and diagnosis may be clinically complex (1, 2). Severe adhesive disease may also markedly distort pelvic anatomy and impair follicular monitoring or oocyte retrieval. In patients with confirmed tubo-ovarian abscesses, systemic inflammatory indices such as the systemic immune-inflammation index (SII) and neutrophil-to-lymphocyte ratio (NLR) have recently been investigated as predictors of medical-treatment failure and the need for invasive intervention (3). However, these findings cannot be directly extrapolated to patients without evidence of active pelvic infection. Hydrosalpinx requires particular consideration before IVF because hydrosalpingeal fluid can adversely affect implantation. In patients with confirmed hydrosalpinx, salpingectomy or proximal tubal occlusion is generally favored when technically feasible, whereas aspiration has less certain durability and may be followed by fluid recurrence (4–7). These recommendations should not be automatically extrapolated to large pelvic cystic lesions of uncertain origin. Image-guided drainage is an established minimally invasive option for selected fluid collections when surgery is difficult or associated with substantial morbidity (8). Ultrasound-guided aspiration of pelvic inflammatory cystic masses before ovulation induction for IVF has also been described (9). We report a patient with secondary infertility, severe pelvic anatomical distortion, and large multiloculated pelvic cystic lesions in whom ultrasound-guided continuous catheter drainage was used as a temporary bridging strategy to improve ovarian visualization and accessibility before IVF.

## 2 Case description

### 2.1 Patient information and clinical findings

A 35-year-old woman presented in March 2022 with a 12-month history of secondary infertility. She had previously delivered a healthy female infant by cesarean section in April 2010. Her medical history also included surgical repair of intussusception during infancy in 1987. The patient reported no fever, lower abdominal pain, abnormal vaginal discharge, or other symptoms suggestive of active pelvic infection. Abdominal examination revealed a soft, non-tender abdomen without a palpable mass. Speculum examination showed no abnormal vaginal discharge or visible cervical lesion. Bimanual examination was limited by discomfort but revealed no obvious adnexal tenderness or cervical motion tenderness. The subsequent drainage procedure was undertaken to improve ovarian visualization and accessibility for IVF rather than for symptom relief.

### 2.2 Diagnostic assessment

Transabdominal gynecologic ultrasonography performed in January 2022 demonstrated an 8.0 cm × 7.0 cm × 5.1 cm uterus with heterogeneous myometrial abnormalities interpreted clinically as compatible with adenomyosis (Supplementary Figure S1). Transabdominal ultrasonography also showed an 8.01 cm × 3.82 cm × 5.98 cm cystic structure in the left pelvic cavity and an 18.40 cm × 8.82 cm × 11.80 cm multiloculated cystic lesion extending between the pelvic organs and mesentery (Figure 1). These lesions markedly distorted the pelvic anatomy. The left ovary could not be separately visualized because it was obscured by the cystic lesions, whereas the right ovary was displaced superiorly and only two antral follicles were initially visible. The patient had no history of ovarian surgery or oophorectomy. Therefore, the left ovary was considered anatomically present but sonographically non-visible before drainage rather than anatomically absent. Archived ultrasonographic images demonstrated predominantly fluid-filled multiloculated lesions with good acoustic transmission. Cyst-wall thickness had not been formally measured during the original examination. Retrospective proportional assessment using the displayed image scale suggested an approximate wall thickness of 2–4 mm. The internal contents did not demonstrate the homogeneous low-level echoes typically associated with a conventional ovarian endometrioma. Color or power Doppler vascularity of the cyst walls and septa was not systematically assessed during the original examination and could not be reliably reconstructed retrospectively.

**Figure 1:**
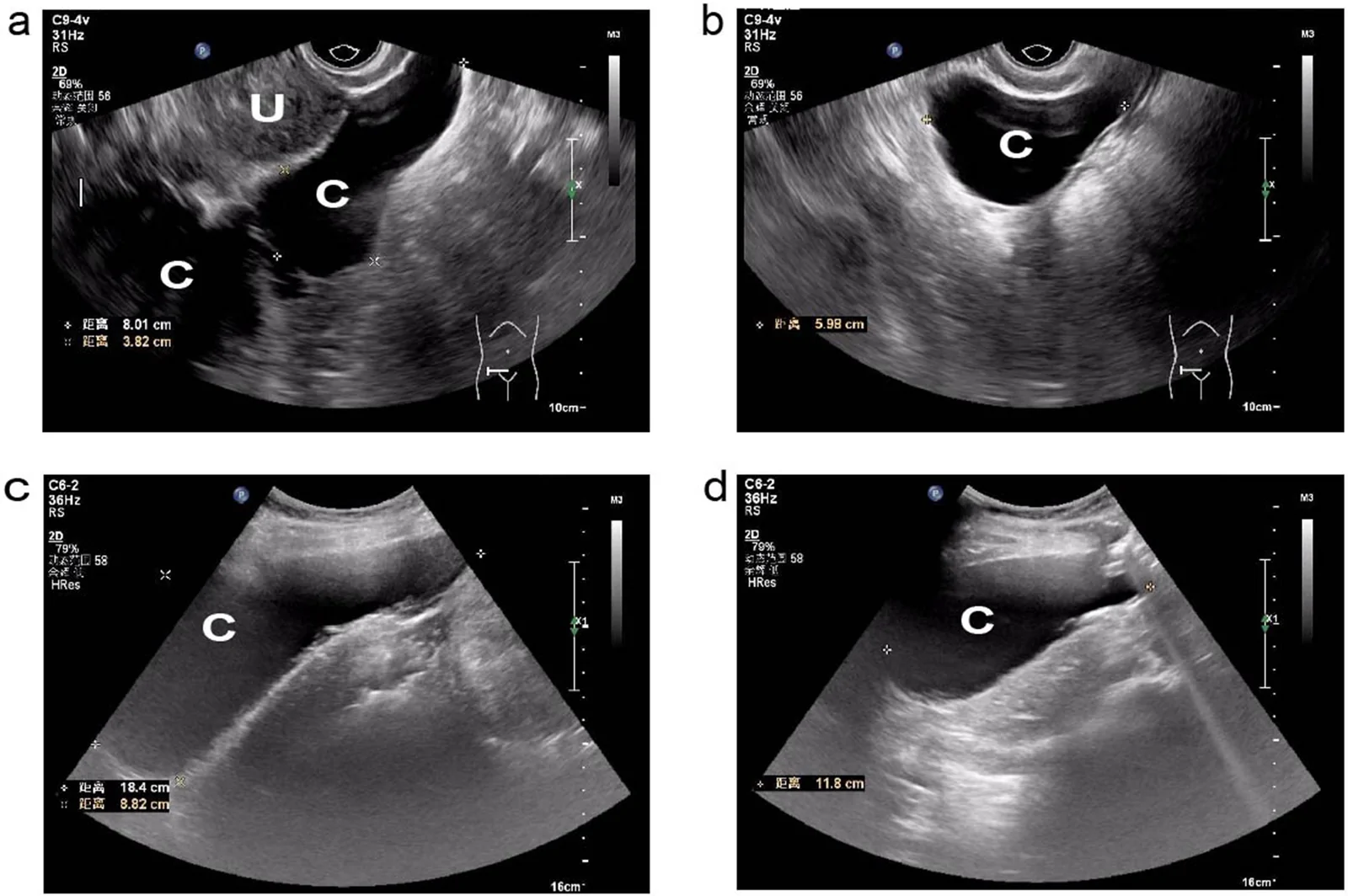
Transabdominal ultrasonography shows a complex pelvic mass system. **(a,b)** 8.01 cm × 3.82 cm × 5.98 cm cystic structure in the left pelvic cavity. **(c,d)** 18.40 cm × 8.82 cm × 11.80 cm multiloculated mass. C, cystic cavity; U, uterus.

Hysterosalpingography demonstrated bilateral tubal occlusion. Complete blood count was normal, C-reactive protein was <0.50 mg/L, Chlamydia trachomatis testing was negative, and vaginal secretion examination showed no abnormality. Serum CA125 was elevated at 82.29 U/mL. Given the concomitant sonographic findings compatible with adenomyosis, this result was interpreted as nonspecific; CA125 could neither establish the origin of the pelvic cystic lesions nor exclude ovarian neoplasia. HE4 was not measured. Serum anti-Müllerian hormone was 1.89 ng/mL. Cycle-day-3 endocrine measurements were as follows: luteinizing hormone, 4.22 mIU/mL; follicle-stimulating hormone, 5.96 mIU/mL; progesterone, <0.16 nmol/L; testosterone, 0.43 nmol/L; prolactin, 260.80 mIU/L; and estradiol, 107.00 pmol/L. These ovarian-reserve parameters suggested that the initially low sonographically visualized antral follicle count underestimated the number of detectable follicles because of anatomical displacement and obscuration.

The precise etiology of the pelvic cystic lesions remained uncertain. Hydrosalpinx was considered because hysterosalpingography demonstrated bilateral tubal occlusion; however, a definite tubular configuration, fallopian-tube origin, or communication between the large lesions and the tubes could not be demonstrated. Therefore, the lesions could not be definitively classified as hydrosalpinges. An active tubo-ovarian abscess was considered unlikely because the patient had no fever, pelvic pain, adnexal tenderness, leukocytosis, elevated C-reactive protein, or purulent drainage. Nevertheless, the absence of active inflammatory findings did not exclude previous pelvic inflammatory disease. A peritoneal inclusion cyst or chronic inflammatory pseudocyst was considered clinically plausible because of the patient's previous abdominal surgery and cesarean delivery, severe pelvic adhesive disease, multiloculated morphology, good acoustic transmission, and subsequent drainage of clear fluid. A typical ovarian endometrioma was considered less likely because the lesions lacked characteristic homogeneous low-level internal echoes and subsequently yielded clear, odorless fluid. Nevertheless, endometriosis could not be definitively excluded without surgical or histopathological assessment. Paraovarian cysts, postoperative fluid collections, simple ovarian cysts, and neoplastic lesions were also considered. Magnetic resonance imaging was not performed, HE4 was unavailable, and no histopathological diagnosis was obtained. Accordingly, the working diagnosis was presumed chronic inflammatory pelvic sequelae with severe pelvic adhesive disease and complex pelvic cystic lesions, rather than definitively confirmed PID-related cystic disease.

### 2.3 Therapeutic intervention

The patient's partner had teratozoospermia, with 3% normal sperm morphology according to Kruger strict criteria. Given bilateral tubal occlusion together with male-factor infertility, IVF was recommended. Definitive surgical management, including adhesiolysis, lesion excision, salpingectomy, or proximal tubal occlusion, was considered. However, the lesions had not been definitively demonstrated to represent hydrosalpinges, and the patient's previous abdominal surgery and cesarean delivery suggested substantial adhesive surgical risk. Additional surgery was therefore deferred because of concern regarding adjacent-organ injury, further adhesion formation, possible ovarian injury, and delay of fertility treatment (4–7). Because the patient was asymptomatic and the immediate clinical problem was mechanical impairment of ovarian visualization and accessibility, image-guided decompression was selected as a temporary bridging strategy.

A 12-Fr pigtail catheter was inserted into the dominant cystic cavity through a transabdominal approach under ultrasonographic guidance (Figure 2). The catheter remained in place for approximately 10 days. Daily drainage decreased from approximately 50 mL on day 1 to <10 mL by day 8, with a total drainage volume of approximately 450 mL. The drained fluid was clear and odorless. Microbiological culture, antimicrobial susceptibility testing, cytological examination, and biochemical analysis of the drainage fluid were not performed. The dominant lesion decreased from 18.40 cm × 8.82 cm × 11.80 cm before drainage to approximately 3 cm × 2 cm at catheter removal. The catheter was removed after daily drainage remained <10 mL for three consecutive days and ultrasonography confirmed collapse of the cystic cavity. The abdominal skin at the catheter insertion site was disinfected with povidone-iodine solution every 2–3 days while the catheter remained in place. Oral levofloxacin 0.5 g once daily was administered from catheter placement until catheter removal, for approximately 10 days, as prophylaxis against secondary catheter-related infection. The choice reflected the treating clinicians’ judgment and was not intended as treatment for active PID. Current recommendations for acute PID emphasize broad-spectrum antimicrobial coverage, including anaerobic coverage; therefore, the regimen used in this patient should not be interpreted as a recommended treatment regimen for acute PID (10). Complete blood counts and C-reactive protein levels were monitored every 3–4 days during catheter drainage and after catheter removal and remained within normal limits. No catheter dislodgement, obstruction, clinically apparent bleeding, local infection, or symptomatic pelvic infection occurred. Following decompression, the total number of sonographically visualized antral follicles increased from two to eight, with five in the left ovary and three in the right. This finding was interpreted as improved visualization of previously obscured follicles rather than an increase in ovarian reserve. The left ovary, which had previously been non-visible, also became accessible.

**Figure 2:**
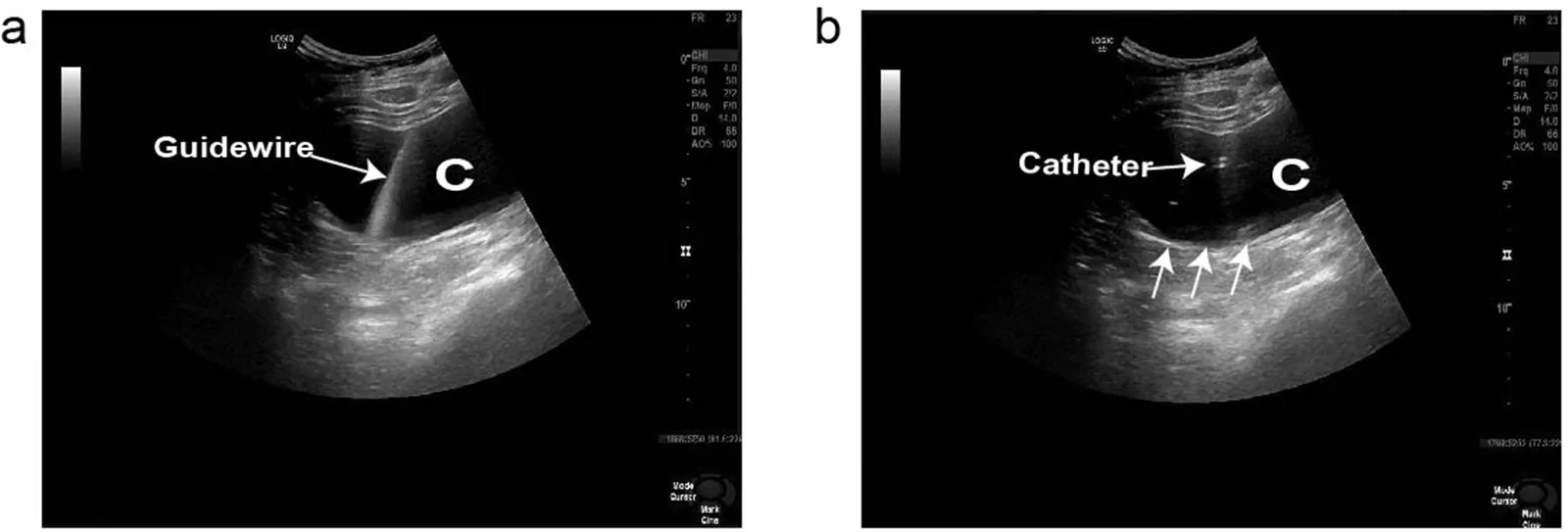
**(a)** A 12-Fr pigtail catheter was inserted via the transabdominal approach over a guidewire. **(b)** The catheter in the cystic cavity. C, cystic cavity; arrowheads indicate the cyst wall.

Controlled ovarian stimulation was initiated on cycle day 3 using recombinant follicle-stimulating hormone 225 IU/day. Ganirelix 0.25 mg/day was administered on stimulation days 7–11, followed by dual triggering with triptorelin 0.2 mg and human chorionic gonadotropin 4,000 IU 36 h before oocyte retrieval. Because the principal pretreatment limitation was anatomical accessibility rather than a documented inadequate ovarian response, this single case cannot determine whether an alternative stimulation protocol would have altered the outcome. At oocyte retrieval, both ovaries were successfully accessed. Five metaphase II oocytes were retrieved, including four from the previously non-visible left ovary and one from the right ovary. Four high-quality blastocysts were subsequently vitrified. For the frozen-thawed embryo transfer cycle, pituitary downregulation was performed with leuprolide acetate 3.75 mg, followed by endometrial preparation with estradiol valerate 4 mg/day for 14 days. Micronized progesterone 600 mg/day and dydrogesterone 40 mg/day were initiated 5 days before transfer. A single frozen-thawed blastocyst was transferred on May 12, 2022.

### 2.4 Follow-up and outcomes

Clinical pregnancy was confirmed on June 12, 2022. Subsequent routine prenatal examinations showed no major abnormalities. At 38 weeks of gestation, repeat cesarean delivery was performed in January 2023 because of breech presentation in the setting of a previous cesarean scar. Intraoperatively, severe pelvic adhesive disease was observed, including dense adhesions involving the peritoneum, uterus, anterior abdominal wall, and bladder. During adhesiolysis required to expose the uterus, an approximately 3.0-cm bladder perforation occurred. The drainage catheter had been removed several months earlier, and there was no intraoperative evidence of catheter-related injury. Previous abdominal surgery, prior cesarean delivery, and severe adhesive disease were considered the most plausible contributors to the difficult dissection, although the relative contribution of individual factors cannot be determined from a single case. The bladder injury was repaired by a urologist using primary cystorrhaphy. An indwelling urinary catheter was maintained for 14 days, and immediate postoperative recovery was uneventful.

No substantial re-accumulation of the dominant cystic lesion was identified during ovarian stimulation, after oocyte retrieval, or on the day of embryo transfer. Routine obstetric ultrasonography did not identify recurrence during pregnancy. The cesarean delivery and cystorrhaphy lasted approximately 150 min. A male infant weighing 2,730 g was delivered. At the postoperative follow-up visit at our hospital in April 2023, the patient reported no notable discomfort. Transabdominal ultrasonography demonstrated no apparent abnormalities in bladder size or internal echoes; both ovaries were clearly visualized, with the right ovary remaining superiorly displaced and measuring approximately 3.18 cm × 1.25 cm, and the left ovary measuring approximately 2.57 cm × 1.90 cm; no significant cystic fluid collection was observed within the pelvic cavity (Figure 3). Whether preoperative urological assessment or additional urinary-tract imaging could have prevented the injury cannot be determined retrospectively. Nevertheless, the complication highlights the importance of anticipating urinary-tract involvement and considering multidisciplinary operative planning when dense bladder adhesions are suspected. At the 12-month clinical follow-up, the patient reported no recurrent pelvic symptoms. The clinical timeline of the case is summarized in Table 1.

**Figure 3:**
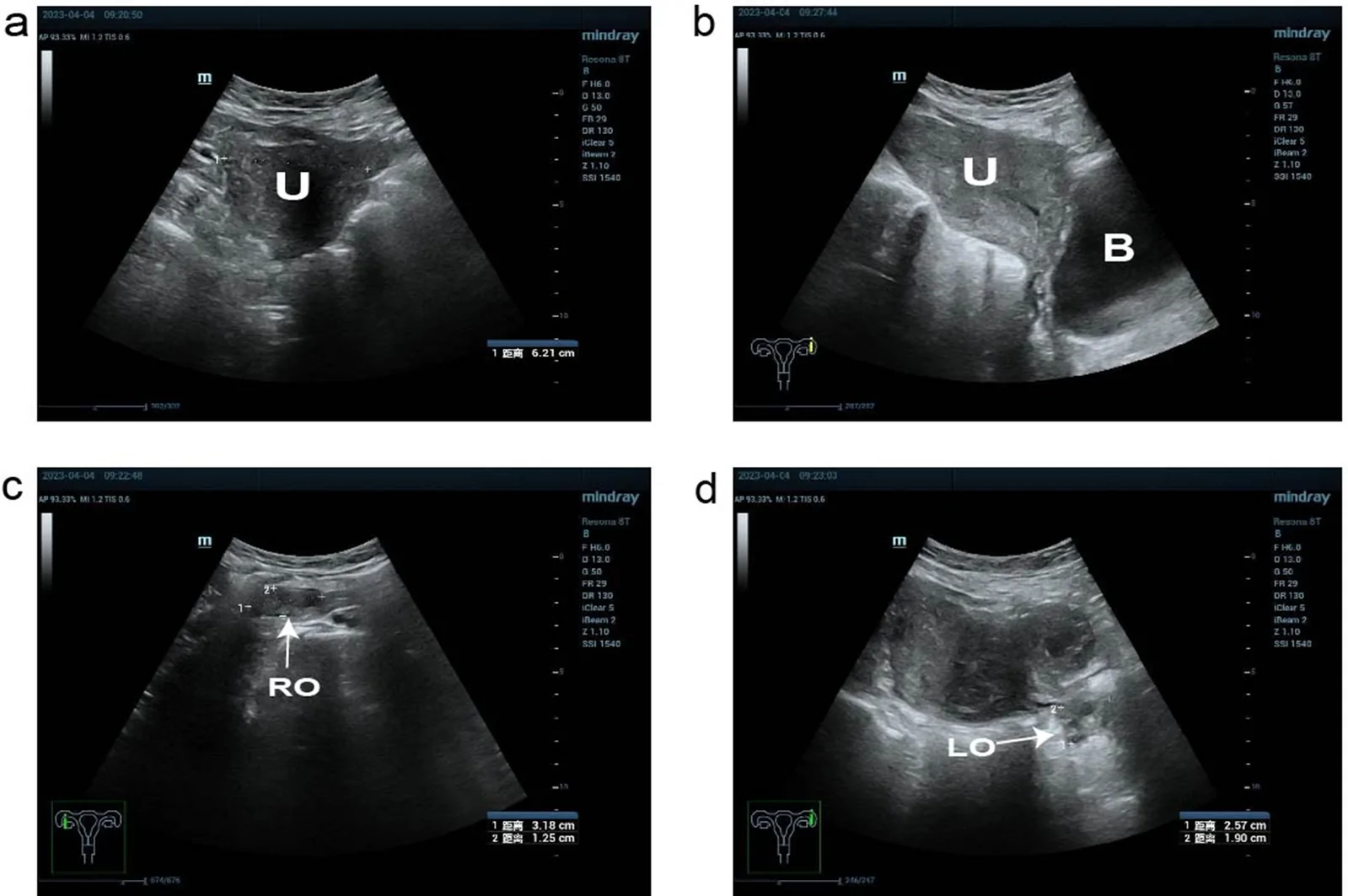
Postoperative follow-up transabdominal ultrasonography shows uterus and bladder **(a,b)**, both ovaries visible:right ovary superiorly displaced 3.18 cm × 1.25 cm and left ovary 2.57 cm × 1.90 cm **(c,d)**. U, uterus; B, bladder; RO, right ovary; LO, left ovary.

**Table 1:** Key events from diagnosis to 12-month clinical follow-up.

Date	Event
January 2022	TVUS demonstrated large multiloculated pelvic cystic lesions; the left ovary was obscured and the right ovary displaced superiorly. HSG demonstrated bilateral tubal occlusion
March 2022	Evaluation for secondary infertility; IVF planned
March 2022	Transabdominal ultrasound-guided placement of a 12-Fr pigtail catheter; approximately 450 mL drained over approximately 10 days
After drainage	Ovarian visualization and accessibility improved; sonographically visualized AFC in the ovary increased from 2 to 8
March 2022	Controlled ovarian stimulation and oocyte retrieval: 5 MII oocytes retrieved (4 left, 1 right); 4 high-quality blastocysts vitrified
May 12, 2022	Single frozen-thawed blastocyst transfer
June 12, 2022	Clinical pregnancy confirmed
January 2023	Repeat cesarean delivery at 38 weeks for breech presentation and previous cesarean scar; 3.0-cm bladder injury repaired by cystorrhaphy
April 2023	Postoperative follow-up: transabdominal US showed normal bladder and both ovaries visible (right 3.18 cm × 1.25 cm, superiorly displaced; left 2.57 cm × 1.90 cm), no pelvic cystic fluid collection.
January 2024	12-month clinical follow-up; no recurrent pelvic symptoms reported

## 3 Discussion

This case illustrates a mechanical challenge in assisted reproduction: severe pelvic adhesive disease and large cystic lesions can make anatomically present ovaries difficult to visualize and access. The principal observation is therefore not that drainage increased ovarian reserve or improved IVF success, but that temporary decompression restored sufficient ovarian accessibility to permit follicular monitoring and oocyte retrieval. This interpretation is particularly supported by retrieval of four of the five mature oocytes from the left ovary, which had not been visible before drainage. Diagnostic uncertainty remains central to this report. The combination of bilateral tubal occlusion, severe adhesions, and multiloculated cystic lesions was compatible with chronic inflammatory pelvic sequelae, but there was no documented episode of acute PID and no microbiological or histopathological confirmation (1, 2). The available imaging and drainage characteristics provided additional but non-definitive information. Good acoustic transmission, retrospectively estimated thin walls, and clear, odorless drainage fluid made a typical ovarian endometrioma or active tubo-ovarian abscess less likely. A peritoneal inclusion cyst or chronic inflammatory pseudocyst was clinically plausible, particularly given the previous abdominal surgery and extensive adhesions, but could not be confirmed.

The distinction between the present lesions and confirmed hydrosalpinx is clinically important. Hydrosalpingeal fluid can adversely affect IVF outcomes, and evidence supports salpingectomy or proximal tubal occlusion before IVF when communicating hydrosalpinx is demonstrated (4–7). In this patient, bilateral tubal occlusion was present, but the large lesions could not be definitively shown to arise from or communicate with the fallopian tubes. Continuous catheter drainage should therefore not be considered equivalent to definitive hydrosalpinx treatment. If recurrent or communicating hydrosalpinx is demonstrated before a future embryo transfer, definitive tubal management should be reconsidered while balancing the substantial surgical risk associated with this patient's adhesive disease.

The present strategy also differs from simple needle aspiration. Image-guided catheter drainage can provide sustained decompression of selected fluid collections (8), while previous IVF literature described ultrasound-guided needle aspiration of pelvic inflammatory cystic masses before ovarian stimulation (9). Here, temporary continuous catheter drainage of a very large multiloculated lesion was used primarily to restore ovarian accessibility. Systemic inflammatory indices such as SII and NLR may help predict medical-treatment failure in confirmed tubo-ovarian abscesses (3), but their applicability to this patient is limited because she had no evidence of active infection or confirmed tubo-ovarian abscess. Accordingly, drainage was undertaken for anatomical rather than infectious indications. Infection nevertheless remained a procedural concern because an indwelling pelvic catheter can provide a route for secondary infection. No clinically apparent infection developed, and serial complete blood counts and C-reactive protein levels remained normal. However, lack of drainage-fluid culture and use of a non-standardized prophylactic antibiotic regimen preclude conclusions regarding optimal infection-prevention strategies.

Recurrence is also clinically relevant. Catheter drainage decompresses a cavity but does not remove its wall, eliminate a possible secretory source, or definitively treat hydrosalpinx if present. Although decompression persisted during the IVF treatment period and no recurrence was detected on routine obstetric or postoperative imaging, the short-term decompression effect in this case was sufficient to support IVF treatment rather than to achieve a persistent anatomical repair. The bladder perforation encountered during repeat cesarean delivery represents an important adverse event. Although it occurred several months after catheter removal and there was no evidence of direct catheter-related injury, it illustrates the potential surgical consequences of severe pelvic adhesive disease. Preoperative recognition of distorted anatomy, involvement of experienced pelvic surgeons, and selective urological consultation may be appropriate when dense bladder adhesions are anticipated.

Several limitations should be acknowledged. First, this is a single case report and cannot establish efficacy, safety, or generalizability. Second, the underlying etiology of the pelvic cystic lesions was not definitively determined. Third, cyst-wall thickness was estimated retrospectively rather than measured prospectively, and Doppler vascularity was unavailable. Fourth, HE4, MRI, histopathological examination, and drainage-fluid culture, cytology, and biochemical analysis were not performed. Fifth, the apparent increase in AFC represented improved sonographic visualization rather than a proven change in ovarian reserve. Sixth, the drainage, catheter-care, and antimicrobial prophylaxis protocols were not standardized. Future prospective studies should standardize imaging characterization, catheter technique, drainage duration, fluid analysis, antimicrobial prophylaxis, catheter-removal criteria, and recurrence surveillance. Comparative studies are also required to determine which carefully selected patients may benefit from temporary image-guided decompression rather than definitive surgery.

### 3.1 Take-away lessons

In this patient, ultrasound-guided continuous catheter drainage functioned as a temporary mechanical bridge that improved access to ovaries obscured or displaced by large pelvic cystic lesions. It should not be interpreted as proven therapy for PID-related infertility, as a method to increase ovarian reserve, or as a substitute for definitive treatment of confirmed hydrosalpinx.

## Patient perspective

The patient reported that the physical difficulties caused by the pelvic cystic lesions made her worry that she might not be able to have another child. She wished to avoid another pelvic operation because she was concerned that repeat surgery might cause further harm. After the clinical team explained that temporary catheter drainage might decompress the pelvic lesions, improve ovarian accessibility, and make ovarian stimulation and oocyte retrieval possible, together with the potential risks and uncertainties of the procedure, she agreed to the proposed treatment. She reported no obvious discomfort during the drainage and IVF treatment process. After subsequently giving birth to a healthy infant, she stated that she was very happy with the outcome.

## Data Availability

The raw data supporting the conclusions of this article will be made available by the authors, without undue reservation.

## Supplementary material

Supplementary Figure S1 Transabdominal ultrasonography shows myometrium lesions. **(a)** 3.95  × 3.20 cm myometrium lesion on the posterior wall. **(b)** 4.90  × 3.82 cm myometrium lesion on the left wall. **(c,d)** 1.79  × 1.40 cm and 3.21  × 2.30 cm myometrium lesions on the anterior wall. U, uterus; M, myometrial lesion; C, cystic cavity.
